# Duodenal fluid analysis of 13 patients with progressive familial intrahepatic cholestasis type 2 from a single institution

**DOI:** 10.1097/MD.0000000000050696

**Published:** 2026-09-18

**Authors:** Benping Zhang, Shengxuan Liu, Biao Zou, Sainan Shu, Chen Dong

**Affiliations:** a Department of Endocrinology, Tongji Hospital, Tongji Medical College, Huazhong University of Science and Technology, Wuhan, Hubei, China; b Department of Pediatrics, Tongji Hospital, Tongji Medical College, Huazhong University of Science and Technology, Wuhan, Hubei, China.

**Keywords:** bile acid, bile salt export pump, cholestatic diseases, duodenal fluid, progressive familial intrahepatic cholestasis type 2

## Abstract

Progressive familial intrahepatic cholestasis type 2 (PFIC2) is an autosomal recessive disease caused by homozygous or compound heterozygous mutations in the bile salt export pump (BSEP). The diagnosis has mainly depended on genetic tests. Little research has been conducted to assess the BSEP function, which may help the clinical diagnosis. The present study aimed to quantitatively assess the BSEP function by collecting duodenal fluid of patients with PFIC2. This is a single-center retrospective study. The clinical presentations, laboratorial and genetic data of 13 patients with PFIC2 were collected for analysis. Eight patients with progressive familial intrahepatic cholestasis 1 and 13 with idiopathic neonatal cholestasis were chosen as the 2 control groups. The diagnostic values of total bile acid in duodenal fluid (dTBA) and dTBA/total bile acid in serum (sTBA) ratio on PFIC2 were assessed. DTBA was significantly lower in patients with PFIC2 than in those with progressive familial intrahepatic cholestasis 1 and idiopathic neonatal cholestasis. The dTBA/sTBA ratio was also significantly lower in patients with PFIC2. Both dTBA and dTBA/sTBA showed high diagnostic sensitivity, specificity, positive and negative predictive values according to the receiver operating characteristic curve analysis. The present study found that duodenal tube test would help to understand PFIC2 and quantitatively access the function of BSEP.

## 1. Introduction

Progressive Familial Intrahepatic Cholestasis (PFIC), a group of diseases caused by bile secretion defects, is characterized by intrahepatic cholestasis in infancy or childhood.^[1]^ So far, twelve types of PFIC (progressive familial intrahepatic cholestasis 1 [PFIC1]~ progressive familial intrahepatic cholestasis 2 [PFIC2]) have been proposed according to genetic testing.^[2]^ In clinical practice, 2 groups were divided according to blood gamma-glutamyl transferase (GGT) levels roughly. Since genetic testing is time consuming, patients with different types of PFIC still present a challenge in the differential diagnosis.

PFIC2 is one of the types with normal or lower than normal GGT level. It is an autosomal recessive disease caused by homozygous or compound heterozygous mutations in the bile salt export pump (BSEP, ABCB11),^[3]^ which is located on the apical membrane of hepatocytes. It is essential for the transport of bile acids from hepatocytes into bile capillaries. The impaired function of BSEP results in the accumulation of bile acids within hepatocytes.

The main clinical manifestations of PFIC2 include cholestasis and severe pruritus in infancy or early childhood. Some patients may progress rapidly to end-stage liver disease and require liver transplantation. So far, PFIC2 is still a disease with limited therapeutic options. Maralixibat, a selective inhibitor of the ileal bile acid transporter, has been used in treatment of PFIC2. The mechanism of ileal bile acid transporter is occluding bile acid return to bloodstream from enteric cavity. However, response to the drug is dependent on the disease subtype.^[4]^ Patients wouldn’t show sustained reductions in serum bile acid levels except for those with non-truncating mutation of BSEP.^[5]^ Therefore, it is necessary to look for new therapies for PFIC2.

Since BSEP acts as a significant role in PFIC2, it is worth developing a method for BSEP function assessment, which may help the diagnosis and treatment of PFIC2.^[6–8]^

Duodenal tube test (DTT) is a test, which reflects bile characteristics, usually performed by gastroenterologists. The test, mainly performed in Chinese and Japanese centers to assess the color and composition of the bile, has been used as a qualitative and quantitative evaluation method to rule out biliary atresia in recent years. Nonetheless, there has been little research on the duodenal fluid characters of other infantile cholestasis diseases.

The present study was designed to analyze concentrations of duodenal total bile acid (TBA) and other indices associated with cholestasis in combination with serum indices in patients with PFIC2 and assess its diagnostic value of PFIC2.

## 2. Methods

This study was approved by the Institutional Ethics Committees of Tongji Hospital, Tongji Medical College, Huazhong University of Science and Technology. The medical records of patients with PFIC2 from May 1, 2013 to April 30, 2022 were collected and reviewed. Informed consent in writing was obtained from each patient’s legal guardian. The study was conducted in accordance with the principles outlined in the Declaration of Helsinki.

### 2.1. Patients

This study included 13 patients with ABCB11 gene mutations admitted to the Pediatrics Department of Tongji Hospital, China, between May 1, 2013 to April 30, 2022. All the 13 patients underwent DTT, and were chosen as the experimental group (e.g.) for further analysis. Mutations in the ABCB11 gene are shown in Table 1. Furthermore, 8 patients with PFIC1 and thirteen patients with other neonatal cholestasis were chosen as the 2 control groups (CG1, CG2). The etiology of patients in CG2 included neonatal hepatitis (n = 4), parenteral nutrition-associated cholestasis (n = 3), cytomegalovirus hepatitis (n = 2), neonatal intrahepatic cholestasis caused by citrin deficiency (n = 2), and Alagille syndrome (n = 2). Patients with biliary atresia or bile acid synthesis defects were excluded.

**Table 1:** Mutations in the ABCB11 gene of the 13 patients with PFIC2*.

Patient	Genotypes			Effects
	Patient (Het†/Homo‡)	Father (Het†/Homo‡)	Mother (Het†/Homo‡)	
1	c.1331T > C/c.1091T > C (Het)	c.1331T > C/wild (Het)	c.1091T > C/wild (Het)	p. V444A/p. L364P
2	c.257T > C/c.3938T > C(Het)	c.257T > C/wild (Het)	c.3938T > C/wild (Het)	p. M86T/p. L1313P
3	c.1331T > C/c.458T > C (Het)	c.1331T > C/wild (Het)	c.458T > C/wild (Het)	p. V444A/p. L153P
4	c.1331T > C/c.990G > C (Het)	c.1331T > C/wild (Het)	c.990G > C/wild (Het)	p. V444A/p. W330C
5	c.713delG/ c.1331T > C (Het)	c.1331T > C/wild (Het)	c.713delG/wild (Het)	p. G238fsX241/ p. V444A
6	c.1331T > C/ c.1331T > C (Homo)	c.1331T > C/wild (Het)	c.1331T > C/wild (Het)	p. V444A/ p. V444A
7	c.1331T > C/ c.1331T > C (Homo)	c.1331T > C/wild (Het)	c.1331T > C/wild (Het)	p. V444A/ p. V444A
8	c.1331T > C/ c.1331T > C (Homo)	c.1331T > C/wild (Het)	c.1331T > C/wild (Het)	p. V444A/ p. V444A
9	c.1445A > G/ c.1331T > C (Het)	c.1445A > G/ wild (Het)	c.1331T > C/wild (Het)	p. D482G/ p. V444A
10	c.1708G > A/ c.1331T > C (Het)	c.1708G > A/ wild (Het)	c.1331T > C/wild (Het)	p. A570T/ p. V444A
11	c.1160G > A/c.779G > A (Het)	c.1160G > A/ wild (Het)	c.779G > A/ wild (Het)	p. R387H/ p. G260D
12	c.1364G > C/ c.1331T > C (Het)	c.1364G > C/ wild (Het)	c.1331T > C/wild(Het)	p. G455A/ p. V444A
13	c.1384A > C/ c.1763C > T(Het)	c.1384A > C/ wild (Het)	c.1763C > T/wild(Het)	p. S462R/ p. A588V

* Progressive familial intrahepatic cholestasis type 2.

† Heterozygous mutation.

‡ Homozygous mutation.

### 2.2. Data extraction

The clinical history and physical examination were taken before laboratory tests and DDT were performed. The clinical presentation, laboratory findings, outcome of DTT (evaluated by the color and levels of indices associated with cholestasis of duodenal fluid), and genetic results were reviewed retrospectively.

Whole-exome sequencing (WES) and Sanger sequencing were used to finish the genetic tests. The patients and their parents’ peripheral blood was collected firstly for DNA extraction. Then WES was performed based on the NovaSeq 6000 platform after IDT XGen Exome Research Panel was used to capture libraries. The reads were mapped to the human reference genome (GRCh38/hq38). Variations were annotated through ANNOVAR and evaluated according to allele frequencies. WES uncovered potential pathogenic variants, which would be screened according to the American College of Medical Genetics and Genomics guidelines.

As we have previously reported, DTT is a simple, rapid, safe, and cost-effective method compared to other tests.^[9]^ Therefore, in this study, duodenal fluid was obtained using DTT rather than endoscopic duodenal intubation. It was performed with a nasoduodenal tube. If there is not an available nasoduodenal tube, it can be replaced by a properly sized nasogastric tube. All patients fasted for (4.33 ± 0.66) hours before the test under intravenous support. If the patients were crying or irritable before the test, they were sedated orally with chloral hydrate (50 mg/kg). The patients were then placed in a right lateral position and intubated through the right nasal cavity, esophagus, and stomach into the duodenum (approximately 40 cm in total). The final position of the drainage tube was in the middle or lower duodenum, which was confirmed by a radiology test.

For DTT, it was considered bile-positive if yellow fluid was collected in the tube. If the duodenal fluid could not be collected, a discontinuous collection during which the patient could be fed simultaneously with the fluid collection was performed within 24 hours; 3~5 mL of the duodenal fluid was collected for analysis.

Blood and duodenal fluid were collected on the same day and immediately sent to our hospital’s clinical laboratory for analysis. Total bile acid (TBA) level was measured by using enzymatic colorimetry. The GGT level was measured by using the circulating enzymatic method. Both indices were tested using Roche biochemical apparatus. Besides total bile acid (TBA) and other indices associated with cholestasis, TBA in duodenal fluid (dTBA)/serum TBA (sTBA) ratio, and dTBA/serum gamma-glutamyl transpeptidase (sGGT) ratio were examined. The above results were prior to treatment for cholestasis including UDCA.

### 2.3. Statistical analysis

Statistical analysis was performed using SPSS version 17.0 (SPSS Inc, Chicago). Continuous data were presented as mean ± SD. The rate was expressed as a percentage. Chi-square test was used to compare clinical characteristics of different groups of patients. T test was used to compare duodenal and serum indices’ levels in different groups. The cutoff values for different serum and duodenal fluid indices and ratio were determined by receiver operating characteristic (ROC) analysis. *P* < .05 was considered statistically significant.

## 3. Results

Thirty-four patients were enrolled in this study, including 13 patients with PFIC2 (9 males and 4 females with a mean age of 5.51 ± 3.42 months), 8 with PFIC1 (5 males and 3 female with a mean age of 7.33 ± 1.53 months) and 13 with other infantile hepatitis syndrome (IHS, 7 males and 6 females with a mean age of 5.13 ± 1.90 months). Clinical characteristics of the patients were shown in Table 2. All the 13 patients with PFIC2 had a normal past medical and family history. The patients with PFIC2 were all referred to our department due to jaundice. Two of the patients had severe coagulation disorders besides jaundice, ten had severe itching, and ten had hepatomegaly with or without splenomegaly. Four of the patients had congenital heart disease.

**Table 2:** Clinical manifestations of the patients.

	Total (n = 34)	PFIC2* (n = 13)	PFIC1† (n = 8)	IHS‡ (n = 13)	P1§	P2¶
Age at DTT, mo	5.53 ± 2.12	5.52 ± 2.68	6.49 ± 1.36	4.96 ± 3.58	.28	.54
Male	21 (61.8%)	9 (69.0%)	5 (62.5%)	7 (53.8%)	.27	.42
Gestational age	38w5d ± 2w1d	39w3d ± 6d	39w ± 1w1d	37w5d ± 3w	.32	.07
Birth weight (g)	2926 ± 461	3108 ± 335	3012 ± 429	2692 ± 516	.60	.02
Jaundice	34 (100%)	13 (100%)	8 (100%)	13 (100%)	–	–
Pale yellow feces	10 (29.4%)	4 (30.1%)	3 (37.5%)	3 (23.1%)	.78	.66
Pitch	19 (55.9%)	10 (76.9%)	6 (75.0%)	3 (23.1%)	.92	<.01
Hepatomegaly	26 (76.5%)	10 (76.9%)	5 (62.5%)	11 (84.6%)	.49	.62
Splenomegaly	14 (41.2%)	5 (38.5%)	3 (37.5%)	6 (46.2%)	1.00	.69
Coagulation disorder	5 (14.7%)	2 (15.4%)	1 (12.5%)	2 (15.4)	.90	1.0
Liver transplantation	2 (5.9%)	1 (7.7%)	1 (12.5%)	0 (0%)	.76	.31
Congenital heart disease	11 (32.4%)	4 (30.8%)	3 (37.5%)	4 (30.8%)	.78	1.0

* Progressive familial intrahepatic cholestasis type 2.

† Progressive familial intrahepatic cholestasis type 1.

‡ Infantile hepatitis syndrome.

§ PFIC2 compared with PFIC1 patients.

¶ PFIC2 compared with IHS patients.

The mean age of infants with PFIC2 at DTT was 5.52 ± 2.68 months. There was no significant difference between the age of infants with PFIC2 and PFIC1, or between the age of infants with PFIC2 and IHS (5.52 ± 2.68 vs 6.49 ± 1.36, *P* = .28; 5.52 ± 2.68 vs 4.96 ± 3.58, *P* = .54).

TBA in the duodenal fluid (dTBA) was significantly lower in patients with PFIC2 than in PFIC1 and IHS (52.9 ± 44.8 vs 193.4 ± 33.3 μ mol/L, *P* < .01) (52.9 ± 44.8 vs 300.8 ± 572.3 μ mol/L, *P* < .01) (Table 3). Total bilirubin (TB) in duodenal fluid (dTB) (13.3 ± 21.0 vs 141.1 ± 20.6 μ mol/L, *P* < .01) (13.3 ± 21.0 vs 111.4 ± 96.4 μ mol/L, *P* < .01), and direct bilirubin in duodenal fluid (dDB) (8.7 ± 15.4 vs 95.0 ± 19.9 μ mol/L, *P* < .01) (8.7 ± 15.4 vs 81.4 ± 80.8 μ mol/L, *P* < .01) were also significantly lower in patients with PFIC2. There were no distinct differences in serum TB or serum direct bilirubin between patients with PFIC2 and PFIC1, or between patients with PFIC2 and IHS. sGGT levels in PFIC2 were significantly lower than in IHS (37.2 ± 16.4 vs 227.2 ± 173.4 μ mol/L, *P* < .01). In terms of sGGT levels, there was no significant difference between patients with PFIC2 and PFIC1(37.2 ± 16.4 vs 31.8 ± 12.2 μ mol/L, *P* = .066). (Table 3)

**Table 3:** Biochemical characteristics of the patients.

		PFIC2* (n = 10)	PFIC1† (n = 3)	IHS‡ (n = 11)	P1§	P2ǁ
TB¶, μmol/L	sTB	185.9 ± 270.8	184.7 ± 17.5	134.6 ± 143.1	.961	.109
	dTB	13.3 ± 21.0	141.1 ± 20.6	111.4 ± 96.4	<.01	<.01
DB#, μmol/L	sDB	160.3 ± 233.9	150.0 ± 20.7	92.2 ± 104.0	.642	.012
	dDB	8.7 ± 15.4	95.0 ± 19.9	81.4 ± 80.8	<.01	<.01
TBA**, μmol/L	sTBA	187.2 ± 41.6	223.2 ± 40.3	122.9 ± 90.6	.068	<.01
	dTBA	52.9 ± 44.8	193.4 ± 33.3	300.8 ± 572.3	<.01	<.01
GGT††, U/L	sGGT	37.2 ± 16.4	31.8 ± 12.2	227.2 ± 173.4	.397	<.01
	dGGT	133.9 ± 83.9	124.9 ± 8.4	218 ± 273.5	.706	.073

* Progressive familial intrahepatic cholestasis type 2.

† Progressive familial intrahepatic cholestasis type 1.

‡ Infantile hepatitis syndrome.

§ PFIC2 compared with PFIC1 patients.

ǁ PFIC2 compared with IHS patients.

¶ Total bilirubin.

# Direct bilirubin.

** Total bile acid.

†† Gamma-glutamyl transpeptidase.

As shown in Table 4, patients with PFIC2 had a significantly lower dTBA/sTBA ratio (0.262 ± 0.199 vs 0.906 ± 0.928, *P* < .01) (0.262 ± 0.199 vs 1.088 ± 1.626, *P* < .05) than patients with PFIC1 and IHS. Furthermore, dTB/TB in the serum (0.072 ± 0.105 vs 0.792 ± 0.856, *P* < .01) (0.072 ± 0.105 vs 0.521 ± 0.565, *P* < .01) and dDB/DB in the serum (0.051 ± 0.089 vs 0.723 ± 0.669, *P* < .01) (0.051 ± 0.089 vs 0.564 ± 0.530, *P* < .01) were significantly lower in patients with PFIC2 than patients with PFIC1 and IHS. Regards dTBA/ sGGT levels, there was a significant difference between patients with PFIC2 and PFIC1. However, dTBA/ sGGT level in patients with PFIC2 did not differ significantly from the index in patients with IHS.

**Table 4:** Biochemical indices’ ratios of the patients.

		PFIC2* (n = 13)	PFIC1† (n = 8)	IHS‡ (n = 13)	P1§	P2ǁ
DB¶/TB#	sDB/TB	0.857 ± 0.947	0.897 ± 0.833	0.742 ± 0.696	0.180	<0.01
	dDB/TB	0.673 ± 0.949	0.701 ± 0.760	0.803 ± 0.653	0.998	0.522
dTB/sTB		0.072 ± 0.105	0.792 ± 0.856	0.521 ± 0.565	<0.01	<0.01
dDB/sDB		0.051 ± 0.089	0.723 ± 0.669	0.564 ± 0.530	<0.01	<0.01
dTBA**/sTBA		0.262 ± 0.199	0.906 ± 0.928	1.088 ± 1.626	<0.01	<0.05
dGGT††/sGGT		3.914 ± 2.380	3.839 ± 3.857	1.043 ± 0.590	0.589	<0.01
dTBA/ sGGT		2.207 ± 1.877	5.581 ± 7.400	0.723 ± 0.892	<0.01	0.601

* Progressive familial intrahepatic cholestasis type 2.

† Progressive familial intrahepatic cholestasis type 1.

‡ Infantile hepatitis syndrome.

§ PFIC2 compared with PFIC1 patients.

ǁ PFIC2 compared with IHS patients.

¶ Total bilirubin.

# Direct bilirubin.

** Total bile acid.

†† Gamma-glutamyl transpeptidase.

**Figure 1.**
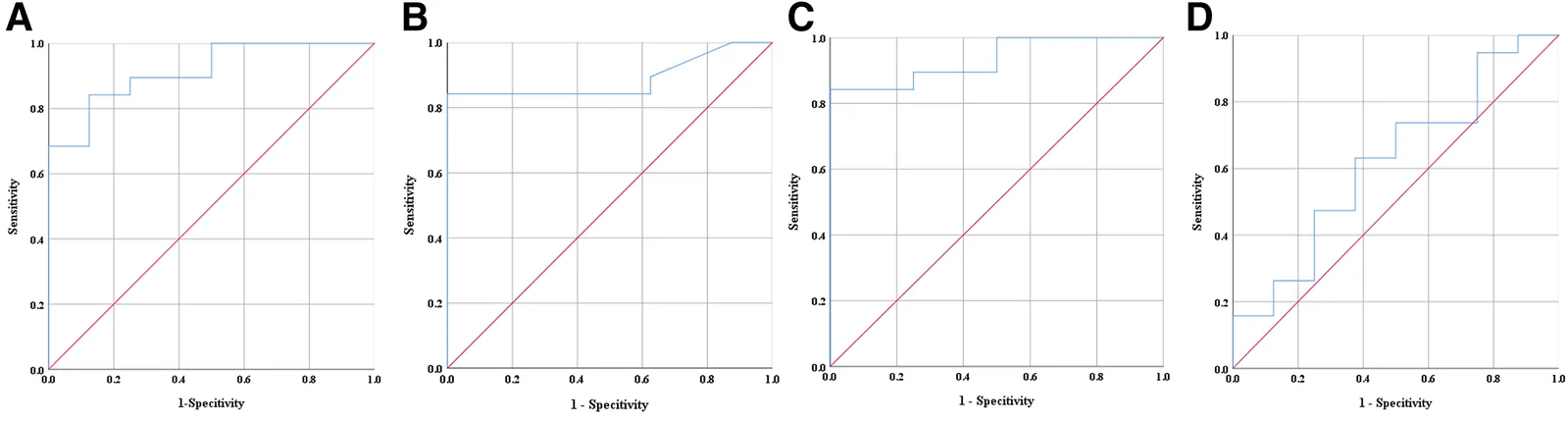
Dot plots of dTBA (A) and dTBA/sTBA ratio (B) in patients with PFIC2 and non-PFIC2. ROC curves of DTT evaluated by dTBA (C) and dTBA/sTBA ((D) PFIC2, progressive familial intrahepatic cholestasis type 2. dTBA = total bile acid in the duodenal fluid, sTBA = serum total bile acid.

The ROC curves for the above parameters are shown in Figure 1. When the dTBA level was <120.80 μ mol/L, the diagnostic sensitivity, specificity, and positive and negative predictive values for PFIC2 were 85.7%, 92.3%, 46.4%, and 52.5%, respectively. The AUC^ROC^ of dTBA was 0.930 (95% confidence interval (95%CI): 0.847–1.000). When the sGGT level was <102.0 μ mol/L, the PFIC2 diagnostic sensitivity, specificity, and positive and negative predictive values were 61.9%, 100%, 50.0%, and 50.0%, respectively. The AUC^ROC^ for sGGT was 0.764 (95% confidence interval (95%CI): 0.603–0.924). The diagnostic sensitivity, specificity, and positive and negative predictive values for PFIC2 were 85.7%, 100%, 55.2%, and 53.8%, respectively, with the optimal cutoff values of 0.717 for dTBA/sTBA. The AUC^ROC^ for dTBA/sTBA was 0.949 (95% confidence interval (95%CI): 0.880–1.000).

## 4. Discussion

Bile acid is synthesized in the hepatocytes, transported into the bile ducts and intestinal tract, reabsorbed by the portal system, and moved back to the hepatocytes again. The process is known as enterohepatic circulation. Through the circulation, approximately 95% of the bile salts are reabsorbed. The circulation is primarily finished by several transporter proteins.^[10]^ BSEP is one of the proteins, mainly responsible for the secretion of bile salts. It predominantly transports monovalent taurine- and glycine-conjugated bile salts from hepatocytes into the bile against a concentration gradient.

Dysfunctional BSEP may result in PFIC2, a cholestatic liver disease that develops in childhood. Genetically, it is characterized by homozygous or compound heterozygous mutations in BSEP. In this study, seventy-seven percent (10/13) of the patients diagnosed as PFIC2 were homozygous or compound heterozygous for V444A. V444A is a common variant of the ABCB11 gene. Despite a small number of studies suggested that the V444A mutation didn’t impair the BSEP function, multiple studies showed the reduced BSEP function with V444A mutation.^[14]^ The latter studies suggested that the mutation is associated with lower BSEP protein levels in human liver samples and in vitro experiments.^[11–13]^ The variant increases the risk of intrahepatic cholestasis of pregnancy and drug-induced cholestasis. The mutation is also linked to the progression of liver cirrhosis with chronic viral hepatitis C.^[14–16]^ In this study, it was found that dTBA and dTBA/sTBA in PFIC2 patients with V444A mutation were significantly lower than normal and other cholestasis diseases, implying the compromised BSEP function.

Jaundice and severe pruritis are the most common clinical manifestations of PFIC2. Untreatable pruritis or liver failure may signal the need for a liver transplantation.^[17–19]^ All the patients diagnosed as PFIC2 (100%) in our study had jaundice. Ten patients (76.9%) had hepatolomegaly, and ten (76.9%) had severe pruritis. One of the patients eventually underwent liver transplantation, who initially presented with severe coagulation dysfunction. The patient’s chief complaints were recurrent petechiae and ecchymosis. It was worth noting that the patient eventually underwent liver transplantation mainly because of the severe and repeated coagulation disorder instead of liver dysfunction.

Several studies have been conducted on the characteristics of serous biochemical indexes, particularly serum GGT and serum TBA levels in patients with PFIC2. In PFIC2 patients, serum GGT levels were normal or lower than normal, with markedly elevated serum bile acid levels.^[3]^ In the present study, both the duodenal fluid and the serum biochemical results of 13 patients with PFIC2 we retrospectively analyzed. Low dTBA and ratios are logical as there is deficiency of BSEP. Through the study, it was further confirmed and showed in detail that dTBA was significantly lower in patients with PFIC2. It was discovered that the sGGT level was significantly lower in patients with PFIC2 than in patients with IHS, which has been verified in previous studies. The sTBA level was significantly higher in patients with PFIC2 than patients with IHS, also consistent with previous findings.^[9]^ Furthermore, it was discovered in this study that the level of dTBA in patients with PFIC2 was significantly lower than that in patients with IHS (Table 3). The reduced dTBA level was due to BSEP dysfunction. BSEP cannot complete the transport of bile in patients with PFIC2. Bile acid accumulates in the hepatocytes and blood. As a result, the bile acid levels in the duodenal fluid were lower than normal. SGGT, dTBA and sTBA all showed significant differences between PFIC2 and IHS patients (Table 3). The above indices make it easy to differentiate between the 2.^[20–22]^

Patients with PFIC1 have normal or lower serum GGT activity and higher sTBA levels. PFIC1 is another common and initially identified type of PFIC. It is an autosomal recessive disease caused by homozygous or compound heterozygous mutations in the ATP8B1 gene, an important transporter that maintains the asymmetry of the inner and outer plasma membranes and protects against high bile salts concentrations.

Indeed, there is no hurry to differentially diagnose between PFIC1 and PFIC2 as management does not change. A secondary result of the study showed that there was significant difference in duodenal fluid between PFIC2 and PFIC1.

The differential diagnosis of PFIC1 and PFIC2 has been difficult due to the similar serum biochemical characteristics. It cannot be completed based on the serum GGT level alone. Several studies have reported that patients with PFIC2 usually have higher serum alanine aminotransferase and alpha-fetoprotein levels compared to PFIC1 patients. It is also reported that patients with PFIC2 have a higher incidence of severe lobular lesions with obvious giant hepatocytes, negative BSEP staining, gallstones, early liver failure, and hepatocellular carcinoma.^[23–25]^ Although the gene test may provide an accurate diagnosis of PFIC1 and PFIC2, the test is expensive and time consuming. In our hospital, the results may be available after 4 to 6 weeks. Therefore, it is worth seeking an accurate, cheap, and quick differential diagnosis method. Of course, there is no urgent need to make a quick differential diagnosis of PFIC1 versus PFIC2 in terms of therapy. It may, however, be useful in determining the carcinogenic risk in patients. Furthermore, when combined with the genetic results, the differential diagnosis may guide the parents while planning for subsequent children. The biochemical characteristics of patients with PFIC1 and PFIC2 were compared in the present study. The dTBA level was found to have a high diagnostic value for PFIC2. As shown in Table 3, there was a significant difference in sGGT levels between PFIC2 and INC infants (*P* < .01); there was, however, no significant difference in sGGT levels between PFIC1 and PFIC2. The dTBA level was significantly lower in patients with PFIC2 than in patients with PFIC1. In addition to dTBA, dTBA/sTBA and dTBA/ sGGT were also significantly lower in patients with PFIC2 than in patients with PFIC1. The mechanism for this difference is primarily due to the different functions of BSEP and ATP8B1.

Furthermore, the ROC analysis revealed that the dTBA, sGGT and dTBA/sTBA levels had similar diagnostic values for PFIC2. Except for sGGT, the other 3 indexes had a higher diagnostic value for differentiating PFIC2 from PFIC1. The indices listed above may aid in the differential diagnosis of PFIC2 and PFIC1.

So far, there have been numerous methods developed to diagnose various cholestasis diseases. DDT has been used mainly to rule out biliary atresia in Chinese and Japanese centers.^[9,26]^ In this study, DDT results were compared between PFIC2 and PFIC1, INC, and normal patients. It was found that DDT could quantitatively assess the BSEP function and had higher diagnostic values for INC, PFIC1, and PFIC2.

In summary, WES is usually used and combined with other tests when necessary in our department. DDT is a quick diagnostic method due to its simplicity. The test has been used to diagnose biliary atresia for many years. Our study found that, in addition to WES et al, bile biochemistry represented various BSEP function states. Combining both may aid in the identification and prognosis of patients with PFIC2. DDT was identified as a useful diagnostic method to differentiate between PFIC1 and PFIC2, which could supplement the previous methods. We are now promoting it as a research tool among patients with enterohepatic circulation disturbance. And in our future work, it will be used to compare between different bile transport disorders (different PFICs).

There are some limitations to the current study. The number of patients was relatively small. Larger studies are needed to confirm these findings.

## Author contributions

**Conceptualization:** Chen Dong, Sainan Shu.

**Data curation:** Shengxuan Liu, Biao Zou.

**Formal analysis:** Shengxuan Liu.

**Methodology:** Biao Zou, Sainan Shu.

**Writing – review & editing:** Chen Dong.

**Writing – original draft:** Benping Zhang.
